# Species-specific duplications driving the recent expansion of NBS-LRR genes in five Rosaceae species

**DOI:** 10.1186/s12864-015-1291-0

**Published:** 2015-02-14

**Authors:** Yan Zhong, Huan Yin, Daniel James Sargent, Mickael Malnoy, Zong-Ming (Max) Cheng

**Affiliations:** College of Horticulture, Nanjing Agricultural University, Nanjing, 210095 China; Centre for Research and Innovation, Fondazione Edmund Mach, San Michele all’Adige, 38010 Italy; Department of Plant Science, University of Tennessee, Knoxville, 37996 USA

**Keywords:** NBS-LRR genes, Rosaceae species, Disease resistance genes, Species-specific duplication

## Abstract

**Background:**

Disease resistance (*R*) genes from different Rosaceae species have been identified by map-based cloning for resistance breeding. However, there are few reports describing the pattern of *R-*gene evolution in Rosaceae species because several Rosaceae genome sequences have only recently become available.

**Results:**

Since most disease resistance genes encode NBS-LRR proteins, we performed a systematic genome-wide survey of NBS-LRR genes between five Rosaceae species, namely *Fragaria vesca* (strawberry), *Malus* × *domestica* (apple), *Pyrus bretschneideri* (pear), *Prunus persica* (peach) and *Prunus mume* (mei) which contained 144, 748, 469, 354 and 352 NBS-LRR genes, respectively. A high proportion of multi-genes and similar *Ks* peaks (*Ks* = 0.1- 0.2) of gene families in the four woody genomes were detected. A total of 385 species-specific duplicate clades were observed in the phylogenetic tree constructed using all 2067 NBS-LRR genes. High percentages of NBS-LRR genes derived from species-specific duplication were found among the five genomes (61.81% in strawberry, 66.04% in apple, 48.61% in pear, 37.01% in peach and 40.05% in mei). Furthermore, the *Ks* and *Ka*/*Ks* values of TIR-NBS-LRR genes (TNLs) were significantly greater than those of non-TIR-NBS-LRR genes (non-TNLs), and most of the NBS-LRRs had *Ka*/*Ks* ratios less than 1, suggesting that they were evolving under a subfunctionalization model driven by purifying selection.

**Conclusions:**

Our results indicate that recent duplications played an important role in the evolution of NBS-LRR genes in the four woody perennial Rosaceae species. Based on the phylogenetic tree produced, it could be inferred that species-specific duplication has mainly contributed to the expansion of NBS-LRR genes in the five Rosaceae species. In addition, the *Ks* and *Ka*/*Ks* ratios suggest that the rapidly evolved TNLs have different evolutionary patterns to adapt to different pathogens compared with non-TNL resistant genes.

**Electronic supplementary material:**

The online version of this article (doi:10.1186/s12864-015-1291-0) contains supplementary material, which is available to authorized users.

## Background

Plants are under constant challenge from a diverse array of pathogens, including bacteria, fungi, oomycetes, viruses and nematodes [1]. Due to high selective pressures, plants have evolved a series of mechanisms to recognize and defend themselves against such pathogens [1,2]. Plant disease resistance (*R*) genes play an important role in pathogen detection and defense response, and are classified into five types, namely NBS-LRR (Nucleotide Binding Sites-Leucine-Rich Repeats), RLK (Receptor-like Kinases), RLP (Receptor-like Transmembrane Proteins), STK (Serine-theorine kinase) and a final category containing all other types of *R* gene. The NBS-LRR genes, encoding nucleotide binding sites (NBS) and leucine-rich repeats (LRR) proteins, are one of the largest plant *R*-gene classes. The NBS domains bind and hydrolyze ATP and GTP, and the LRR motif is involved in protein-protein interactions and pathogen recognition specificity [3]. NBS-LRR proteins can be further sub-divided into two types based on their N-terminal amino acid sequences, TIR-NBS-LRR containing a Toll/Interleukin-1 receptor domain and non-TIR-NBS-LRR genes which contain a coiled-coil (CC), or leucine zipper motif [4].

The major function of NBS-LRR genes is disease resistance through pathogen recognition [1,2]. Patterns of rapid *R*-gene evolution could help host plants recognize avirulence genes and activate downstream transduction cascades, leading to defense responses, hypersensitive reactions and programmed cell death [1]. Abundant NBS-LRR genes have been identified in various plants, e.g. *Arabidopsis*, *Medicago*, *Vitis* (grapevine), *Populus* (poplar), *Oryza sativa* (rice) and *Zea mays* (maize) [5-9]. Interestingly, distinct numbers of NBS-LRR genes were found among these species [5-9]. Since all plant *R*-genes are assumed to have originated from one common ancestor [10], the various gene numbers might therefore be derived from gene duplications in a given species. Recent studies have shown that the NBS gene family possesses a higher proportion of duplicate genes than other gene families [8,11]. The rapid gene expansion or contraction of this family might therefore be a survival strategy to combat rapidly-evolving, species-specific pathogens [6,8].

The Rosaceae family is an economically-important family throughout the world, and comprises many important fruits, such as strawberries, apples, pears, peaches, meis and apricots, as well as flowers and ornamental trees such as roses and rowans [12]. Various pathogens infect these plants leading to a variety of crop diseases, such as powdery mildew, scab, fire blight and sharka disease. Serious economic losses from such pathogens have highlighted the necessity of disease resistance breeding in Rosaceae crops [13,14], and as such the *R*-genes from many members of the Rosaceae family have previously been studied for the purposes of resistance breeding [13-17]. Nevertheless, classic genetic analysis is difficult on Rosaceae species, because many of them are woody perennial plants with a long intergeneration time and a large plant size. Although some powdery mildew, scab and fire blight resistance genes have been identified and mapped in various Rosaceae species, such as the apple scab resistance gene *Rvil5*/*Vr2* encoding TNL proteins, and a fire blight resistance gene encoding CNL protein in *Malus × robusta* [18-27], a genome-wide analysis of Rosacese *R-*genes would permit the identification of additional resistance genes for marker-assisted selection.

The recent completion of genome sequences of the woodland strawberry (*F. vesca*) [28], apple (*M. ×domestica*) [29], Asian pear (*P. bretschneideri*) [30], peach (*P. persica*) [31] and mei (*P. mume*) [32] provides the opportunity to study the evolution of NBS-LRR genes between five Rosaceae genomes. In this work, various numbers of genes and gene families were identified across the five species. Subsequently, different evolutionary patterns of TNL genes and non-TNL genes were observed. Our results suggest that recent, species-specific duplications may have contributed to the rapid expansion of NBS-LRR genes in these species.

## Results

### NBS-LRR genes in five Rosaceae species

A total of 144, 748, 469, 354 and 352 NBS-LRR genes were detected in the *F. vesca*, *M. ×domestica*, *P. bretsvhnrideri*, *P. persica* and *P. mume*, genomes, respectively (Table 1). All Rosaceae species contained more non-TIR-NBS-LRR genes (non-TNLs) than TIR-NBS-LRR genes (TNLs) (Table 1). However, unlike non-TLNs, the proportion of TNLs in strawberry (15.97%) was lower than those in the other four species (29.28% in apple, 47.12% in pear, 36.16% in peach and 43.47% in mei, Table 1). Non-TNLs were further classified into CC-NBS-LRR (CNL) and X-NBS-LRR (XNL) genes, and similar gene numbers for each class were observed in the five species, except in pear. In terms of the relative proportions of the different R-gene classes in the five genomes, the same smallest and largest relative values were found in the strawberry and apple genome, respectively (Table 1).

**Table 1:** **The NBS-LRRs in genomes of five Rosaceae fruit species**

**Predicted protein domains**	***Fragaria vesca***	***Malus domestica***	***Pyrus bretschneideri***	***Prunus persica***	***Prunus mume***
**NBS-LRR genes**	**144**	**748**	**469**	**354**	**352**
TIR-NBS-LRR	23 (15.97%)	219 (29.28%)	221 (47.12%)	128 (36.16%)	153 (43.47%)
non-TIR-NBS-LRR	121 (84.03%)	529 (70.72%)	248 (52.88%)	226 (63.84%)	199 (56.53%)
CC-NBS-LRR	60	243	160	108	99
X-NBS-LRR	61	286	88	118	100
Whole genome genes	32831	57386	42767	27852	31390
Proportion of NBS-LRR genes	0.44%	1.30%	1.10%	1.27%	1.12%
Average exon no. of TIR-NBS-LRR	5.04	7.01	6.21	6.11	6.48
Average exon no. of non-TIR-NBS-LRR	4.83	4.45	3.57	3.09	3.02
Average exon no. of NBS-LRR genes	4.86	5.2	4.81	4.18	4.52
Average exon no. of all genes	5.09	4.74	4.7	4.97	4.6

The mean number of exons revealed in NBS-LRR genes was 4.86 in strawberry, 5.2 in apple, 4.81 in pear, 4.18 in peach and 4.52 in mei. The average exons were lower than observed for all predicted genes in strawberry, peach and mei, but greater than those observed in apple and pear. The average exon number of TNLs was greater than those in non-TNLs in the genomes of all five species, consistent with observations made in the grape and poplar genomes [7], and 1.04-, 1.58-, 1.74-, 1.98-, 2.15-fold were detected between the average exon numbers of TNLs and non-TNLs in strawberry, apple, pear, peach and mei, respectively.

### Gene families of NBS-LRR genes in five Rosaceae fruit species

Gene duplication have contributed to the high numbers and proportions of NBS-LRR genes in plant families [33]. To explore the duplications in the five Rosaceae species examined here, gene families were defined and detected based on two criteria (Table 2: coverage > 70% and identity > 70%). The greatest and smallest numbers of gene families were found in apple (107) and strawberry (12), respectively. Accordingly, the four woody species possessed greater proportions of multi-NBS-LRR-genes than strawberry (32.64%) which is the only herbaceous plant represented by the five species. In apple (68.98%), pear (63.33%) and peach (65.82%), the proportions of multi-genes comprised over 50% of total NBS-LRR genes in each genome. Using more stringent criteria (>80%) as the measurement for recent duplication, the proportions of multi-genes observed decreased by around 10% compared with that resulted from the less stringent standard (>70%) among all five genomes (Additional file 1: Table S1), but in apple, pear and peach, the proportions were still in excess of 50%. However, when the most stringent criteria (>90%) were used, the proportions of multi-genes reduced significantly in all five species (Wilcoxon signed ranks test, *P* < 0.05).

**Table 2:** **Classification of NBS-LRRs in genomes of five Rosaceae fruit species**

	***F. vesca***	***M. domestica***	***P. bretschneideri***	***P. persica***	***P. mume***
Number of single gene	97	232	172	121	182
Number of multi-gene	47	516	297	233	170
Number of gene family	12	107	86	53	49
Average members per family	3.92	4.82	3.45	4.40	3.47
Proportion of multi-gene	32.64%	68.98%	63.33%	65.82%	48.30%
Number of TNL multi-gene	9	147	137	95	82
Number of TNL gene family	1	30	43	19	23
Proportion of TNL multi-gene	39.13%	67.12%	61.99%	74.22%	53.59%
Number of non-TNL multi-gene	38	369	160	138	88
Number of non-TNL gene family	11	77	43	34	26
Proportion of non-TNL multi-gene	31.40%	69.75%	64.52%	61.06%	44.22%

Additionally, non-TNLs were more abundant in each species than TNLs and were present in largest numbers in both multi-genes and gene families (Table 1). The proportion of multi-genes in both non-TNLs and TNLs corresponded with the data observed for NBS-LRR genes in the five species. However, the number of gene families and the proportion of multi-genes between the two types of NBS-LRR genes were distinct between the different genomes; non-TNLs displayed lower proportions of multi-genes than TNLs in strawberry, peach and mei, whereas larger proportions were found in apple and pear, consistent with the closely evolutionary relationship between the latter two species. Therefore, the results suggested that non-TNLs have undergone more recent duplication than TNLs in the two *Maloideae* species, apple and pear. From various perspectives, NBS-LRR genes showed distinct duplication history not only in different species but also in different gene types.

### Evolutionary history of NBS-LRR genes in five Rosaceae fruit species

**Figure 1:**
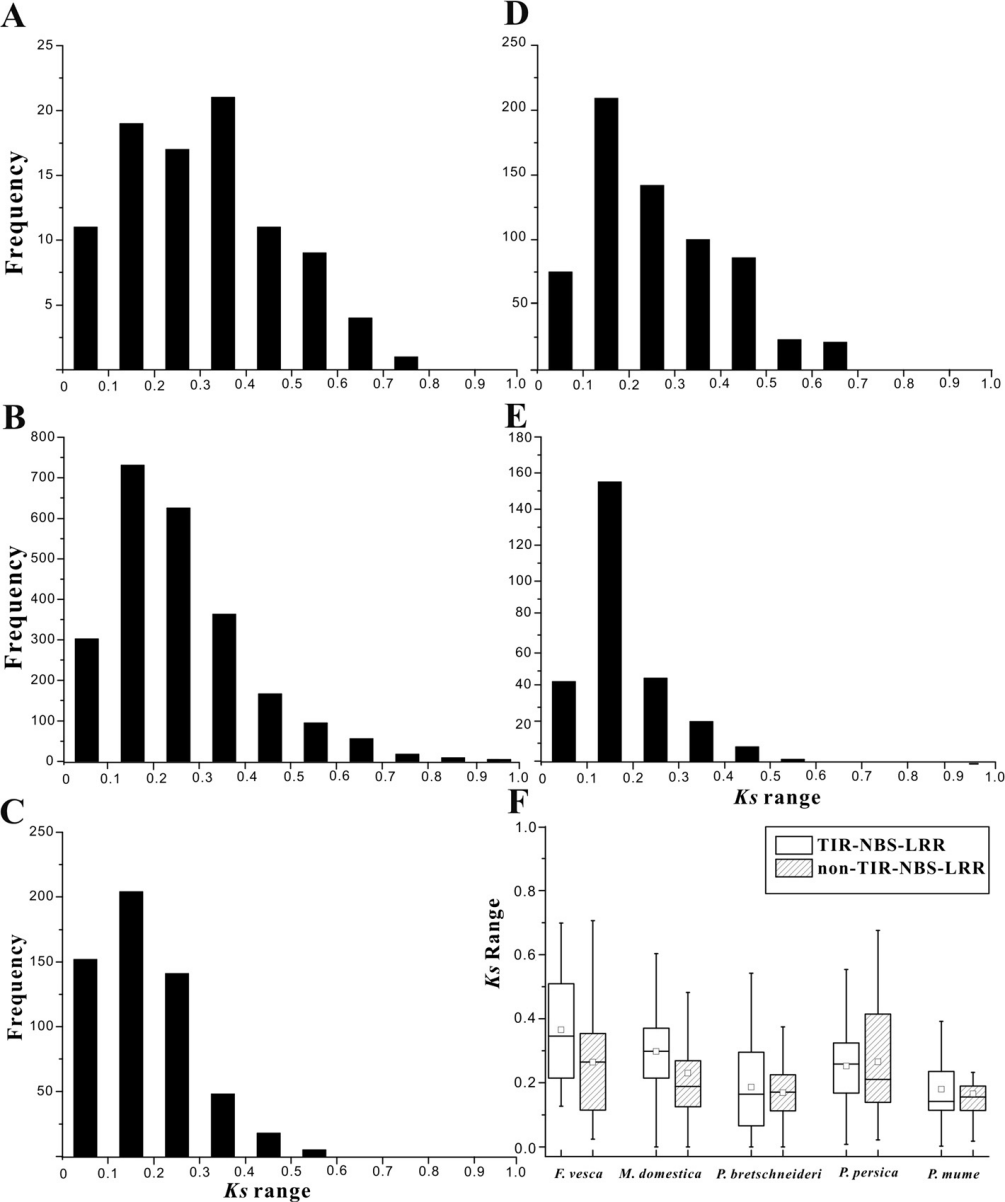
**The** ***Ks*** **values of NBS-LRRs in five Rosaceae fruit species. A-E**: the distribution of *Ks* frequency in the genomes of strawberry, apple, pear, peach and mei, respectively; **F**: The *Ks* ranges of TNLs and non-TNLs in the five species. The bars at the top and bottom of the whiskers mean maximum and minimum values; the top and bottom of the box represent third and first quartiles; the square and bar in the box mean average and median values.

The *Ks* value is commonly used as molecular clock to measure the time elapsed since gene duplication [34]. To explore the duplication time of NBS-LRRs in each of the five genomes, the average *Ks* value of each gene family (Table 2) was calculated. *Ks* values exhibited continuous distribution in all five genomes, suggesting NBS-LRR gene duplication is an ongoing process in each species. However, the distribution of *Ks* was distinctly different among five species, especially between the herbaceous strawberry (Figure 1A) and the four ligneous plants (Figure 1B-E). The relatively even distribution of *Ks* values at the range of 0 to 0.4 with a slight peak at 0.3 to 0.4 in strawberry genome suggests that duplication events have been a relatively constant process. The *Ks* values in the four ligneous species peaked at the range of 0.1 to 0.2 then decreased slowly from 0.2 to 0.8, indicating the relatively recent duplication of NBS-LRR genes in those species.

Figure 1F shows the different distribution patterns of the *Ks* values exhibited between TNLs and non-TNLs in each species. The TNLs had broader distributions of *Ks* than non-TNLs in the genomes of apple, pear and mei, indicating that TNLs had longer duplication process than non-TNLs in these three species. The larger median and quartile ranges suggests that the *Ks* values of TNLs were significantly greater than those of non-TNLs (Mann–Whitney U test) in strawberry (*P* < 0.01) and apple (*P* < 0.01), demonstrating that the corresponding TNL genes underwent duplication at different times in the two species.

### Phylogenetic analysis of NBS-LRR genes

**Figure 2:**
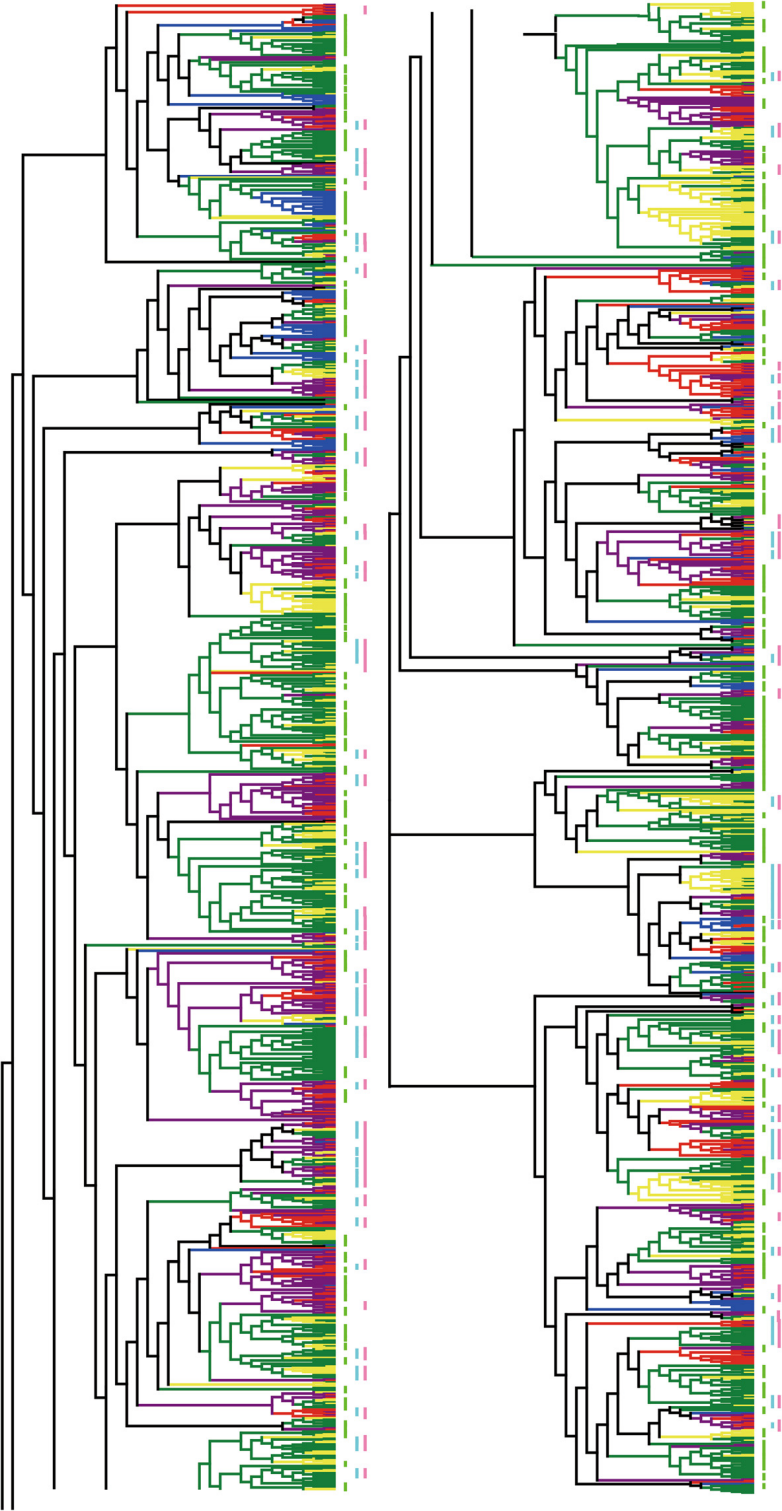
**Phylogenetic tree of NBS-LRRs in the genomes of five Rosaceae fruit species.** The blue, green, yellow, red and purple branches represent genes from strawberry, apple, pear, peach and mei, respectively; pink and sky blue line indicate lineage-specific duplication and species-specific duplication events in it, respectively; light green line means species-specific duplication. The tree was artificially divided into two parts because of space limitation.

To further understand the duplication patterns of NBS-LRR genes in the five genomes, an unrooted phylogenetic tree was constructed based on the nucleotide sequences of the core NBS domain by FastTree 2 (Figure 2). The phylogenetic tree showed few Rosaceae-wide large duplication clusters despite the close evolutionary relationship between taxa, and the topology of genes was consistent with the relationship of species (Figure 2).

When the ML tree was divided into clades according to the bootstrap values (>50), two types of clades which resulted from species-specific duplication (Figure 2: vertical light green lines) and lineage-specific duplication (Figure 2: vertical pink lines) could be clearly observed. The former were denoted as gene duplications of NBS-LRR only occurring in one species, while the latter were composed of gene duplications in more than one species (Figure 2). Within the lineage-specific expansions, many species-specific duplication events (vertical sky blue lines) could also be found. There were 233 species-specific duplication clades and 152 lineage-specific duplication clades with 1083 species-specific duplicated NBS-LRR genes, which further indicated that duplication was the main contributor to the large amount of NBS-LRR genes in each of the five Rosaceae species. There were 650 genes in species-specific duplication clades (vertical light green lines) and 433 genes in species-specific duplication events in lineage-specific duplication clades, demonstrating that 52.39% (1083/2067) of the total NBS-LRR genes were involved in species-specific duplication in the five Rosaceae species. Among these genes, 89 (98/144 = 61.81%), 494 (494/748 = 66.04%), 228(228/469 = 48.61%), 131(131/354 = 37.01%), and 141 (141/352 = 40.05%) species-species duplication genes were detected in strawberry, apple, pear, peach and mei, respectively. Although NBS-LRR genes in strawberry appeared in only some parts of the phylogenetic tree, a relatively higher proportion of species-specific duplicated genes were found in strawberry genome. In addition, the relatively smaller percentages of species-specific duplication genes in peach and mei still showed that nearly 40% of the NBS-LRRs were generated after the split of the two closely-related *Prunus* species, whilst the remaining NBS-LRRs were directly obtained from the *Prunus* ancestor (Figure 2). Thus, our results indicate that species-specific duplication was the main factor driving NBS-LRR expansion in the five Rosaceae species.

To learn more about the evolutionary relationships between known *R*-genes encoding NBS-LRR proteins, we located them on the phylogenetic tree by BLAST search against whole genome CDSs of apple. Interestingly, the NBS-LRRs resistance to apple scab (*Rvil5* and *Rvi1*) and fire blight (*FB_MR5*) were located in apple-specific or apple/pear-specific duplicate clades (data not shown). The *Rvi5* gene had the highest identify with MDP0000754718, which clustered with MDP0000505414 and MDP0000565527, which together formed a specific-specific duplicate clade of apple. For *Rvi1*, it had highest identity with MDP0000278168, which was located in a lineage-specific duplication clade composed of genes only present in apple and pear, whilst another gene encoding CC-NBS-LRR protein, FB_MR5, resided in an apple-specific duplicate clade.

### Nonsynonymous and synonymous substitution of NBS-LRR genes

The ratio of nonsynonymous to synonymous nucleotide substitution (*Ka*/*Ks*) is used as an important indicator of selective constraint in gene diversification. A *Ka*/*Ks* ratio greater than 1 indicates that genes are driven by positive selection, and a ratio of 1 indicates neutral selection, whereas the ratio less than 1 implies purifying selection. To identify the selective pressures working on NBS-LRR genes in the five Rosaceae genomes, *Ka*/*Ks* ratios were estimated in each gene family by MEGA v5.0 [35].

**Figure 3:**
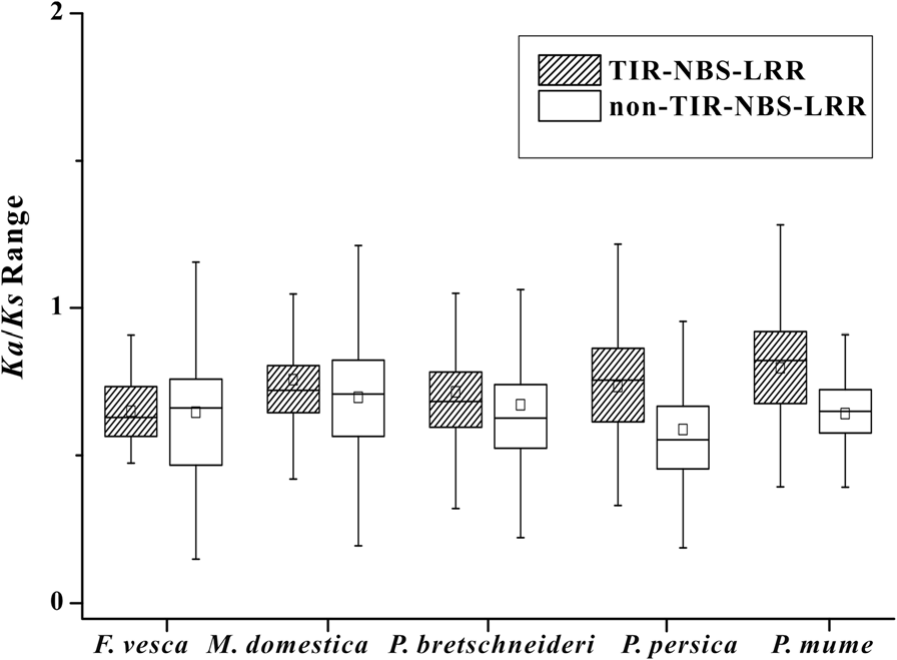
**The** ***Ka*** **/** ***Ks*** **ratios of NBS-LRRs in genomes of five Rosaceae fruit species.**

Figure 3 shows that the majority of the NBS-LRR gene pairs (95.49%) had *Ka*/*Ks* ratios less than 1, including TNLs and non-TNLs, which indicated that most of the genes were driven by purifying selection in the five species. Nevertheless, 3 (0 *vs*. 3), 100 (32 *vs*. 68), 35 (13 *vs*. 22), 23 (9 *vs*. 14) and 17 (15 *vs*. 2) gene pairs had *Ka*/*Ks* ratios greater than 1 for TNLs *vs.* non-TNLs in strawberry, apple, pear, peach and mei, respectively, demonstrating that some of NBS-LRR genes were under positive selection or relaxed selection for gene pairs with *Ka*/*Ks* approximately equal to 1. Among all gene pairs, 0 (0 *vs*. 0), 34 (7 *vs*. 27), 8 (4 *vs*. 4), 7 (5 *vs*. 2) and 6 (6 *vs*. 0) pairs had *Ka*/*Ks* ratio approximately equal to 1 for TNLs *vs.* non-TNLs in strawberry, apple, pear, peach and mei, respectively, indicating that these genes might have undergone mutations causing nonfunctionalization or pseudogenization. The distribution ranges of *Ka*/*Ks* ratios between non-TNLs were broader than those between TNLs according to the lengths of the boxes and whiskers in strawberry, apple and pear (Figure 3). Conversely, *Ka*/*Ks* had narrower distribution between non-TNL genes than TNL genes in the two *Prunus* species (Figure 3). In spite of the differences in distribution ranges, TNLs had larger median and quartile values than non-TNLs in all five species. Except for the values in strawberry, the other *Ka*/*Ks* ratios of TNLs and non-TNLs showed highly significant differences (Mann–Whitney U test, *P* < 0.01) in apple, pear, peach and mei. Interestingly, all TNLs had significant higher *Ka*/*Ks* ratios than non-TNLs, suggesting that TNLs evolve faster and are under stronger selective pressures than non-TNLs.

### Selective pressures of NBS-LRRs among five Rosaceae species

To further detect the evolutionary fate of NBS-LRR duplicates among the five Rosaceae species, site and branch models were implemented using PAML4 (phylogenetic analysis by maximum likelihood) [36] (Tables 3 and 4). Using the site model test, 168 gene families were estimated which contained three or more members, including 59 TNL gene families (strawberry: 1, apple: 14, pear: 20, peach: 12 and mei: 12) and 109 non-TNL gene families (strawberry: 7, apple: 41, pear: 24, peach: 21 and mei: 16). The 2Δln values supported the phenomenon that there were amino acid sites driven by positive selection in both TNL and non-TNL gene families (Tables 3 and 4). In TNL families, all duplicates examined by LR tests showed that the amino acid sites among them were under highly significant positive selection, except family6 in pear. Similarly, in non-TNL gene families, LR tests showed that 88.99% of them had sites driven by highly significant positive selection. It was suggested that these positively selected sites provided relative high substitutions compared with others among NBS-LRR genes.

**Table 3:** **Selective pressures of TNLs among five Rosaceae species**

**Family no.**	***Ka*** **/** ***Ks*** ^**a**^	**ω** ^**b**^	**2Δln**	**Family no.**	***Ka*** **/** ***Ks*** ^**a**^	**ω** ^**b**^	**2Δln**
***F. vesca***				***P. bretschneideri***			
0	0.65	0.64	178.25^**^	28	0.69	0.65	127.83^**^
***M. domestica***				29	0.49	0.55	26.94^**^
0	0.71	0.72	130.72^**^	32	0.74	0.82	86.53^**^
1	0.78	0.80	169.72^**^	37	0.88	0.92	34.57^**^
3	0.61	0.64	77.86^**^	39	0.73	0.55	70.55^**^
4	0.70	0.70	34.50^**^	***P. persica***			
7	0.70	0.79	39.21^**^	1	0.61	0.58	46.66^**^
9	0.88	1.15	269.70^**^	2	0.78	0.69	200.14^**^
10	0.71	0.65	127.61^**^	3	0.54	0.53	87.14^**^
13	0.53	0.65	151.63^**^	5	0.51	0.47	22.47^**^
14	0.88	0.67	275.29^**^	6	0.56	0.54	12.24^**^
15	0.77	0.68	263.14^**^	7	0.87	0.95	404.73^**^
16	0.78	0.87	289.13^**^	8	0.87	0.82	173.19^**^
23	0.97	1.06	177.42^**^	9	0.81	0.81	132.38^**^
24	0.60	0.61	27.06^**^	11	0.66	0.59	186.11^**^
25	0.74	0.70	155.33^**^	12	0.83	0.65	92.60^**^
***P. bretschneideri***				14	0.78	0.73	60.48^**^
2	0.54	0.57	292.28^**^	16	0.84	0.82	49.62^**^
3	0.69	0.75	169.17^**^	***P. mume***			
4	0.65	0.76	143.59^**^	0	1.15	1.31	257.80^**^
6	0.72	0.74	6.79^*^	2	0.89	1.03	333.07^**^
7	0.80	0.86	108.95^**^	4	0.52	0.58	18.40^**^
10	0.65	0.69	127.79^**^	6	0.80	0.81	173.58^**^
11	0.88	0.93	161.28^**^	8	0.97	1.02	125.15^**^
14	0.65	0.67	125.28^**^	10	0.60	0.51	30.89^**^
16	0.92	0.92	259.19^**^	11	0.56	0.54	132.21^**^
17	0.78	0.88	35.67^**^	12	0.89	0.94	344.19^**^
19	0.86	0.99	142.46^**^	14	0.92	0.94	313.67^**^
21	1.08	1.25	130.03^**^	16	0.75	0.71	99.29^**^
22	0.69	0.65	196.90^**^	19	0.79	0.77	49.99^**^
25	0.56	0.47	51.69^**^	20	0.89	0.92	127.40^**^
26	0.64	0.60	62.54^**^				

^a^Average *Ka*/*Ks* ratio of each gene family calculated by MEGA; ^b^ *dN*/*dS* ratio for each gene family using branch model; 2Δln represents the result of LR test for site model; ^*^ and ^**^ represent significant (2Δln > 5.991, *p* < 0.05) and highly significant (2Δln > 9.210, *p* < 0.01) tests for positive selection between model M7 and M8.

**Table 4:** **Selective pressures of non-TNLs among five Rosaceae species**

**Family no.**	***Ka*** **/** ***Ks*** ^**a**^	**ω** ^**b**^	**2Δln**	**Family no.**	***Ka*** **/** ***Ks*** ^**a**^	**ω** ^**b**^	**2Δln**
***F. vesca***				***P. bretschneideri***			
0	1.01	1.37	244.51^**^	12	1.13	1.14	174.51^**^
2	0.51	0.51	30.00^**^	13	0.67	0.64	309.43^**^
3	0.72	0.78	163.67^**^	17	0.69	0.72	270.23^**^
4	0.52	0.52	26.42^**^	19	0.61	0.71	140.67^**^
5	0.35	0.39	3.41	21	0.71	0.88	173.72^**^
9	0.57	0.57	64.46^**^	23	0.62	0.62	69.89^**^
10	0.52	0.53	30.72^**^	24	0.96	0.96	101.27^**^
***M. domestica***				25	0.66	0.67	75.95^**^
0	0.34	0.37	20.55^**^	26	0.51	0.65	30.17^**^
2	0.75	0.65	34.70^**^	27	1.70	0.68	38.52^**^
3	0.78	0.71	164.45^**^	28	0.60	0.67	174.99^**^
6	0.55	0.58	47.64^**^	29	1.21	1.19	124.18^**^
8	0.75	1.38	330.32^**^	32	0.62	0.64	40.06^**^
9	0.40	0.46	11.37^**^	33	0.35	0.40	63.30^**^
11	0.64	0.64	26.32^**^	38	0.73	0.75	426.85^**^
13	0.55	0.58	3.41	40	0.20	0.28	1.27
14	0.88	0.79	53.12^**^	42	0.43	0.50	49.36^**^
15	0.87	0.45	157.04^**^	***P. persica***			
16	0.54	0.58	128.15^**^	0	0.67	0.72	90.19^**^
17	0.65	0.59	45.42^**^	1	0.48	0.46	94.92^**^
20	1.10	1.08	90.23^**^	2	0.58	0.59	64.94^**^
22	1.02	1.33	48.89^**^	3	0.51	0.57	81.35^**^
23	0.59	0.61	123.77^**^	4	0.73	0.85	81.38^**^
24	0.53	0.65	17.75^**^	5	0.56	0.60	53.61^**^
25	0.91	0.95	242.51^**^	7	0.87	0.77	131.61^**^
27	0.55	0.62	236.80^**^	10	0.54	0.56	39.07^**^
29	0.70	0.84	46.93^**^	11	1.01	1.12	35.54^**^
30	0.54	0.59	5.43	12	0.53	0.51	161.31^**^
32	0.41	0.49	3.81	13	0.33	0.38	0.18
35	0.89	0.62	141.09^**^	14	0.59	0.59	68.99^**^
36	0.60	0.70	61.85^**^	15	0.62	0.77	11.16^**^
37	0.65	0.71	30.67^**^	16	0.72	0.87	149.36^**^
39	0.85	0.95	182.68^**^	17	0.64	0.64	94.83^**^
41	0.67	0.70	133.44^**^	20	0.48	0.53	28.45^**^
43	0.93	0.86	158.52^**^	22	0.37	0.39	1.92
46	0.76	0.76	88.84^**^	23	0.53	0.61	17.38^**^
48	0.31	0.47	2.97	24	0.48	0.48	57.74^**^
49	0.70	0.91	18.54^**^	29	0.62	0.62	156.86^**^
51	0.73	0.86	118.63^**^	30	0.68	0.69	86.74^**^
52	0.84	0.86	88.35^**^	***P. mume***			
53	0.25	0.32	1.41	0	0.66	0.74	26.03^**^
54	-	0.00	0	2	0.73	0.72	118.53^**^
56	0.91	1.04	100.03^**^	3	0.68	0.73	173.81^**^
57	0.81	0.91	230.38^**^	5	0.68	0.73	214.41^**^
59	0.74	0.70	278.72^**^	6	0.67	0.70	49.62^**^
60	0.74	0.66	273.76^**^	8	0.70	0.69	101.58^**^
70	0.82	0.78	208.35^**^	10	0.64	0.62	97.65^**^
72	0.40	0.34	61.52^**^	12	0.59	0.65	103.39^**^
75	0.73	0.55	47.28^**^	14	0.63	0.69	63.55^**^
***P. bretschneideri***				15	0.50	0.48	59.19^**^
0	0.60	0.67	312.45^**^	17	0.82	0.79	156.79^**^
1	0.40	0.47	61.89^**^	18	0.58	0.70	179.70^**^
2	0.70	0.78	113.38^**^	19	0.43	0.49	3.83
4	0.42	0.53	18.57^**^	21	0.31	0.34	−2.00E-06
7	0.55	0.63	16.11^**^	24	0.92	1.21	38.51^**^
9	0.54	0.61	54.18^**^	25	0.78	0.89	15.13^**^
11	0.74	0.88	58.97^**^				

^a^Average *Ka*/*Ks* ratio of each gene family calculated by MEGA; ^b^ *dN*/*dS* ratio for each gene family using branch model; 2Δln represents the result of LR test for site model; ^**^ represent highly significant (2Δln > 9.210, *p* < 0.01) tests for positive selection between model M7 and M8.

For ω values >1, a higher substitution rate is characteristic of neofunctionalization between duplicates according to Ohno’s model [37]; a ω value ≈ 1 suggests a constant loss rate caused by neutral nonfunctionalization; and under subfunctionalization, the loss rate is steady at first, then declines leading to a ω value < 1 [38]. Although most of the genes investigated had positively selected sites, they were still under purifying selection. In total 91.07% of all gene families had average ω ratios smaller than 1 and 7.14% of them were larger than 1. Moreover, there were still some gene families had ω approximately equal to 1, which indicated that they were nonfunctionalized duplicates. It was shown that most of NBS-LRR genes became subfunctionalized duplicates but with relative high substitution between sites among them, supporting the rapid subfunctionalization model followed by neofunctionalization [39].

### Evolutionary analysis of RPW8 domain-containing NBS-LRRs

In *Arabidopsis*, the RPW8 gene contains an RPW8 domain which confers broad resistance to powdery mildew [40]. In strawberry, apple, pear, peach and mei, 24, 20, 22, 11 and 13 NBS-LRR genes respectively, encoded not only NBS domain and LRR motif but also a RPW8 domain (PF05659.6). With the exception of two genes (MDP0000196734 & Pb11_0_1_0), all of the 90 RPW8 domain-containing NBS-LRRs in all five species were non-TNL genes, including 33 CC-NBS-LRR genes and 55 X-NBS-LRR genes.

**Figure 4:**
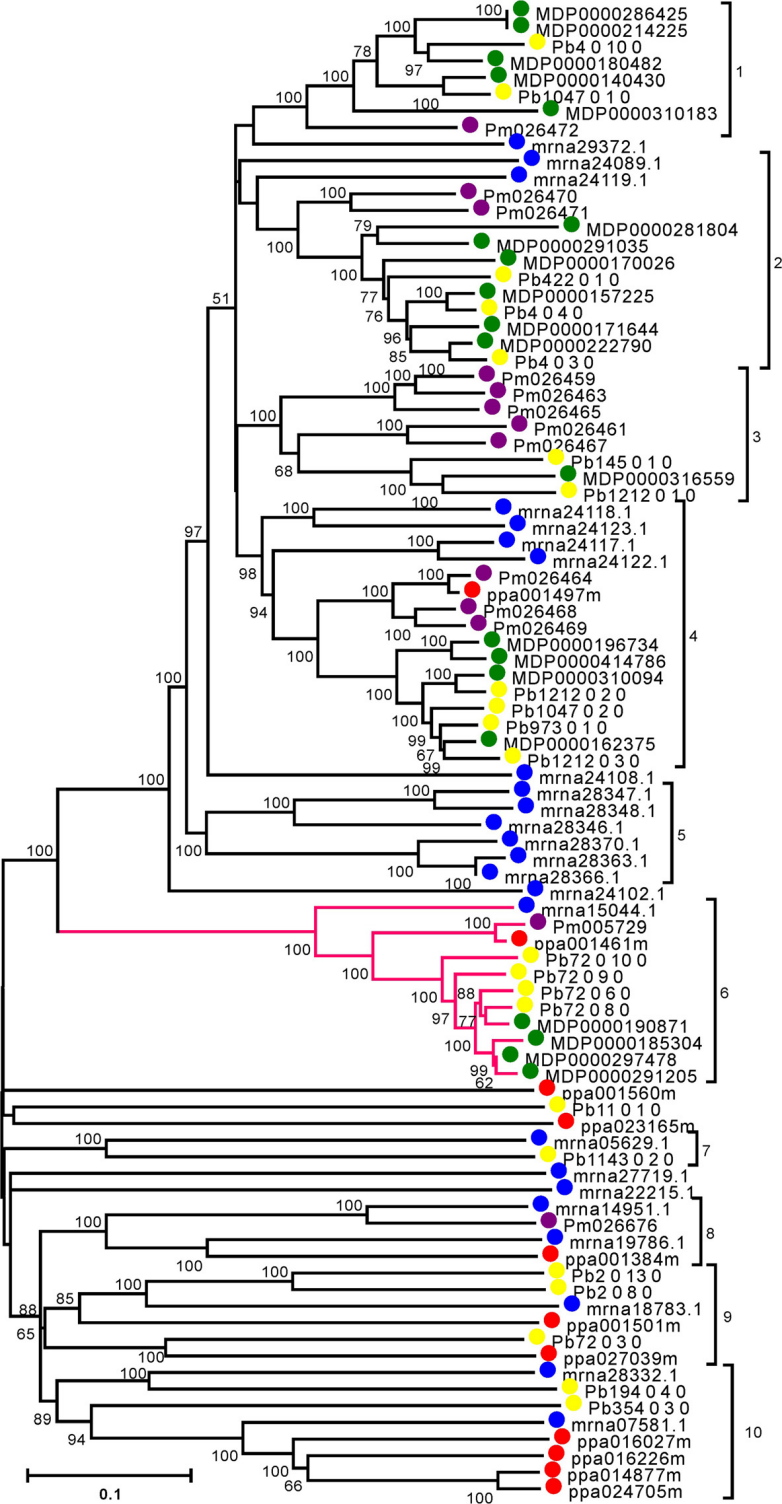
**A phylogenetic tree of RPW8 domain-containing NBS-LRRs in the genomes of five Rosaceae fruit species.** Blue dots: strawberry, green dots: apple, yellow dots: pear, red dots: peach, and purple dots: mei. Red branch represents Group 6 whose members have the highest identity with RPW8 gene.

In the un-rooted phylogenetic tree constructed according to the nucleotide sequences of RPW8 domain-coding regions, ten groups were detected in the tree based on the topology (Figure 4). The RPW8 domain-containing NBS-LRRs exhibited two distinct topologies: a group of genes clustered together with shorter branch lengths (Group 1 to 6), whereas another clustered with longer branch lengths and deeper nodes (Group 7 to 10), indicating that the latter might be the ancestral genes of the former. Interestingly, gene members in Group 6 exhibited relatively high identities compared to the RPW8 genes in *Arabidopsis* using BLAST search, indicating that these genes might be considered as *R*-gene candidates. To investigate the evolutionary pattern and selective constraints, nucleotide diversities and *Ka*/*Ks* ratios for the genes in each group were calculated. Genes in Group 7 to 10 had highly significant nucleotide diversity compared with those in Group 1 to 6 (Additional file 2: Table S2, Mann–Whitney U test, *P* < 0.01) their longer branch lengths demonstrating that these genes showed two different evolutionary patterns. (Additional file 3: Table S3) shows that, with the exception of three gene pairs (MDP0000310183/MDP0000180482, MDP0000310183/ MDP0000286425 and MDP0000310183/ MDP0000214225), all of the gene pairs had *Ka*/*Ks* ratios less than 1, indicating that most of the RPW8 domain-containing NBS-LRRs were under purifying selection. Furthermore, the *Ka*/*Ks* ratios of Group 1 to 6 were higher than those of Group 7 to 10 (0.42 *vs.* 0.37), which demonstrated that former were under stronger selective pressure than latter.

## Discussion

### Recent duplication in NBS-LRR genes

Gene duplication is one of the prevalent forces leading to increased gene numbers and genome complexity in eukaryotes [41-44]. Gene duplication contributes to the evolution of gene networks [45] and provides the raw material for the evolution of novel genetic systems [37] and gene function [46,47].

NBS-LRR genes are one of the largest gene families in plant genomes and the largest class of known disease resistance genes (*R*-genes), which play important roles in response to pathogens [5,7,48,49]. Absolute numbers of NBS-LRR genes varied between the four ligneous species investigated here, but similar relative proportions of NBS-LRR genes were identified as in *A. thaliana* [5]*.* The smaller number and proportion of NBS-LRR genes identified in strawberry (*F. vesca*) might be associated with its smaller genome and its lack of genome-scale duplications that have been identified in other rosids [28]. Additionally, the relative proportions of NBS-LRRs in the four woody genomes analyzed here were similar with those found in the rice genome [7], despite the different absolute number of NBS-LRR genes. Therefore, the number of NBS-LRR proteins was not related to the whole-genome predicted protein numbers. The NBS-LRR genes were mainly (approximately 50%) generated by recent gene duplications in the five Rosaceae species, especially in the four woody species according to relatively strict criteria (coverage > 70% and identity > 70%), and exceeding 50% of NBS-LRR genes were still detected in three woody species based on strict standards (>80%), which were previously used as the proxy for the detection of recent duplication events [7,8]. The *Ks* distributions also support the hypothesis that recent duplication events resulted in the expansion of NBS-LRR genes in the five species, with the proportions of genes in the *Ks* range of 0 to 0.2 being 32.26% (strawberry), 43.57% (apple), 62.68% (pear), 43.29% (peach) and 72.10% (mei). The *Ks* peak at the same narrow range (0.1 ~ 0.2, Figure 1) observed in the four woody species suggests that the corresponding duplications might have occurred at the same period considering these NBS-LRR genes derived from the common ancestor with the similar nucleotide mutation rates. In apple genome, *Ks* of all paralogs peaked around 0.2 for a recent whole genome duplication, which occurred 30–45 MYA [29]. Therefore, it could be inferred that the recent duplication (*Ks* = 0.1 ~ 0.2) of NBS-LRR genes might occurred less than 30–45 MYA.

In addition, duplicate genes most likely face three evolutionary fates: (a) nonfunctionalization or pseudogenization might cause one gene copy to lose function; (b) subfunctionalization partitions ancestral gene function in daughter copies; and (c) neofunctionalization leads to one copy retaining its original function and the other evolving a novel function [50,51]. In our study, it was found that most of NBS-LRR gene families (91.07%) have undergone subfunctionalization (ω < 1), whilst a small number have undergone nonfunctionalization (ω ≈ 1) or neofunctionalization (ω > 1). Although the nonfunctionalization model is thought to be the most common fate of duplicates, previous research supports the subfunctionalization model in the preservation of complex genes after duplication [52]. Among the NBS-LRR genes under subfunctionalization, most of them had positively selected sites with relative high substitution rate, consistent with previous research that subfunctionalized duplicated genes act as a transition state to neofunctionalization, which is a prolonged and substantial process during evolution of gene duplication [39,53].

### Species-specific duplication mainly contributes to NBS-LRR gene expansion

Species-specific duplication is defined as the expansion of a gene family only in one species compared with other species. This phenomenon was observed in this investigation, with clustering of paralogous genes together with each other but not with orthologous genes in phylogenetic trees (Figure 4). This result is in accord with the fact that NBS-LRR genes have been shown to undergo gene expansion after speciation, in species such as *A. thaliana* [5,54], *O. sativa* [48], *Zea mays* [8], *Populus* and *V. vinifera* [7]. In the 101 lineage-specific clades identified, 122 species-specific duplicate clades exhibited a “one-to-one” topology and 263 species-specific duplication events showed a “one-to-many” topology (Additional file 4: Figure S1). The “one-to-many” topology demonstrated that the gene copies inherited from ancestral species were retained in one species, but that they were reserved and expanded into multi-copies in another species after species divergence, in a similar way to what has been reported in four gramineous plants, *Z. mays*, *S. bicolor*, *B. distachyon* and *O. sativa* [8]. In addition, a relatively larger proportion of NBS-LRR genes were counted in strawberry (61.81%), apple (66.04%), pear (48.61%), peach (37.01%), and mei (40.05%) in species-specific duplicates clades of ML tree. This suggests that species-specific duplication led to the major expansion of NBS-LRR genes in the five Rosaceae species studied here. In addition, the existence of lineage-specific or species-specific clades indicates the existence of mechanisms for cluster conservation, as reported by Plocik et al. [55].

Previous studies have shown that species-specific gene duplication leads to species-specific gene functions and features, which could improve the adaptation of the corresponding species to the changing environment [11,56-59]. Although the five species in this study belong to the same family, the different species have markedly different characteristics. The five plants could be divided into two classes, herbaceous and ligneous plants, and the NBS-LRR genes in the only herbaceous plant strawberry, exhibited a different evolutionary pattern and feature from those in the other four ligneous plants. For example, the least absolute number (144), relative proportion (0.44%) and multi-gene proportion (32.64%) of NBS-LRR genes were found in strawberry compared with other plants (Tables 1 and 2) and an even distribution of duplication time (*Ks* value) was only detected (Figure 1) in strawberry. These data indicate that NBS-LRR genes in strawberry experienced a gradual accumulating process to adapt to their specific physiological characteristics and ecological changes. One reason for the higher number of *R*-genes observed in the four woody species compared with strawberry may be that long-lived perennial woody plants face more abiotic or biotic stresses before reproduction [60] or fruit ripening [61]. Therefore, the larger *R*-gene numbers and proportions in four woody species could be considered as disease resistance gene pools to rapidly evolve novel resistance specificities [62]. Furthermore, NBS-LRR genes of the four ligneous plants also show some distinct features from each other, such as the different topologies of NBS-LRRs and species-specific duplicate clades in each genome (Figure 2).

Locating known Rosacous resistance genes, and specifically those of apple in the tree showed that the two apple scab genes (*Rvi1* and *Rvi5*) clustered in two clades specific for apple and pear, and the same result was observed for the CC-NBS-LRR gene of *Malus × robusta* 5 confering the resistance to fire blight (data not shown). These data suggest that different Rosaceae plants have different environment and life histories, thus species-specific *R*-genes are duplicated to response to their specific ecological environment, pathogens or natural selective pressures.

Several clades include a mixture of NBS-LRR genes from *Fragaria, Malus, Pyrus*, and *Prunus*, indicating that similar resistance genes are still shared in different genera of the Rosaceae, which support the monophyletic origin of the four genera. Most of these clades are composed of CNL genes whilst a few contain TNL genes. Moreover, similar resistance gene analogues (RGAs) were shared in *Malus, Pyru*s, and *Prunus*, indicating high conservation of specific RGAs and suggesting a monophyletic origin of these genera as well [63].

### Different evolutionary patterns between TNL and non-TNL genes

NBS-LRR genes with ancient origins can be divided into two types, TNLs and non-TNLs, both of which can be found in gymnosperms and angiosperms [64]. Interestingly, TNLs are present in eudicots, but absent in monocots [65,66]. The data presented here, along with previous studies suggests that TNL and non-TNL genes exhibit different evolutionary patterns [6,11]. In the *A. thaliana* and *A. lyrata* genomes, the branch lengths of TNL genes clades were significantly longer than those of non-TNLs in the phylogenetic tree, indicating that TNL genes might evolve faster than non-TNLs [6]. In this study, the estimation of synonymous and nonsynonymous rates permitted detection of the evolutionary patterns between TNL and non-TNL duplicate genes. The significantly greater *Ks* values of TNLs than those of non-TNLs in strawberry and apple genomes (Mann–Whitney U test, *P* < 0.01) demonstrate that TNL duplicated earlier and had a faster evolutionary rate compared with non-TNL duplicates within the two species, respectively. In addition, the similar tests and phenomenon were also reported in the soybean genome (1.6-fold, *P* < 0.001) [11] and *Arabidopsis* relative genomes (1.4-fold, *P* < 0.05) [6]. Thus, this suggests that TNLs and non-TNLs have different evolutionary rates in responding to co-evolving pathogens.

*Ka*/*Ks* ratios were estimated to detect the diversifying selection pressures on TNL and non-TNL genes. TNLs had more *Ka*/*Ks* ratios greater than 1 compared with non-TNLs. TNLs had significantly greater *Ka*/*Ks* ratios than non-TNLs, consistent with the results detected in soybean genome, suggesting that stronger selective pressure might act on TNLs than on non-TNLs. The rapid evolutionary pattern of TNL genes might be one the strategies used by plants to protect themselves against diverse pathogens. However, in poplar and grapevine, non-TIR NBS families had significantly greater *Ka*/*Ks* ratios than TIR NBS families (*P* < 0.05) [7,67]. These results suggested that different plants with different life histories, thus the diverse evolution of TNLs and non-TNLs, might be driven by specific ecological environments. Moreover, independent evolution of the TNLs and non-TNLs could be detected in the phylogenetic trees of NBS-LRRs from each species (Additional files 4, 5, 6, 7 and 8: Figure S1-S5), demonstrating that the five trees were all divided into two parts: TNLs and non-TNLs, except some XNLs or CNLs located in TNL clades, which was previously suggested in the *V. vinifera* genome [68]. Therefore, TNL genes and non-TNL genes most likely have different evolutionary patterns to adapt to different pathogens.

An interesting domain structure, RPW8-NBS-LRR, was found in 88 non-TNLs among the NBS-LRR genes in the five Rosaceae species studied, including 33 CNL and 55 XNL genes. We infer from this that the novel domain structure might have occurred from the fusion of an RPW8 domain with a CNL [7] or XNL. Furthermore, the similar architecture was also detected in grapevine and poplar, involving five CNLs and one XNL, respectively [7], far fewer than the corresponding gene numbers detected in the five Rosaceae genomes in this investigation (24 in strawberry, 20 in apple, 22 in pear, 11 in peach and 13 in mei). The high number in the five species compared with grapevine and poplar might be a result of specific duplication events that have occurred in the evolutionary history of the Rosaceae to adapt to Rosaceae-specific pathogens. However, there were distinct gene numbers of RPW8-NBS-LRR between the five species, caused by species-specific duplication (Figure 4) to respond to their species-specific functional requirement [11,56-59]. The RPW8 domain is shared with the *Arabidopsis* RPW8 protein exhibiting broad resistance to powdery mildew [40], suggesting that the novel domain structure might provide distinctive function for plant defense system, such as higher powdery mildew resistance, especially the members in Group 6 (Figure 4), which might be the candidate gene for powdery mildew resistance.

In Rosaceae, most of woody perennial plants have a long intergeneration cycle and large plant sizes, which limits rapid breeding for disease resistance in these plants. The selective pressure of NBS-LRR genes in the five Rosaceae genomes demonstrated different selection pressures. Although most of paralogous pairs had *Ka*/*Ks* ratios less than 1, some paralogous pairs had *Ka*/*Ks* ratios greater than 1, indicating that these genes were driven by positive selection. The combination of the genetic relationships of NBS-LRR genes shown in the phylogenetic trees (Figures 2 and 4), the natural selective pressures on those genes and the *R*-genes identified in previous studies, will enable the effective selection of candidate genes for disease resistance breeding in Rosaceae crops.

## Conclusions

Based on a genome-wide survey, we identified 144, 748, 469, 354 and 352 NBS-LRR genes in strawberry, apple, pear, peach and mei, respectively. We found that recent duplications (<30 - 45 MYA) generated the higher proportion of multi-genes and similar *Ks* peaks (0.1- 0.2) in NBS-LRRs of the four woody perennial Rosaceae species. In the phylogenetic tree, we detected species-specific duplication leading to high percentages of NBS-LRR genes in strawberry (61.81%), apple (66.04%), pear (48.61%), peach (37.01%) and mei (40.05%), and suggest that species-specific duplication has mainly contributed to the expansion of NBS-LRR genes in the five Rosacese species. In addition, the *Ks* and *Ka*/*Ks* values of TNLs were significantly greater than those of non-TNLs, indicating that TNLs evolved rapidly with different evolutionary patterns to response to distinct pathogens compared with non-TNLs, and most NBS-LRRs had *Ka*/*Ks* ratios less than 1, demonstrating that they were driven by purifying selection and their evolutionary fates were towards subfunctionalization.

## Methods

### Identification of NBS-LRR genes and classification of the gene family

The whole genome sequences and annotations of woodland strawberry (*F. vesca*) [28], apple (*M. ×domestica*) [29] and peach (*P. persica*) [31] were downloaded from the FTP site of Phytozome v9.0 (ftp://ftp.jgi-psf.org/pub/compgen/phytozome/v9.0). The pear (*P. bretschneideri*) [30] assembled sequences were retrieved from website of the Center for Pear Engineering Technology Research, Nanjing Agricultural University (http://peargenome.njau.edu.cn/), whilst the mei (*P. mume*) [32] genome sequences and annotations were obtained from Beijing Forestry University (http://prunusmumegenome.bjfu.edu.cn/).

The standard NB-ARC domain (PF00931) was first obtained from the Pfam website [69] (http://pfam.sanger.ac.uk/) as the query sequence to BLAST against the whole genome CDSs, and candidate CDSs were generated using TBLASTN searches with an expectation value ≤ 10^−4^ in strawberry, apple, peach and mei. The same standard NB-ARC domain was used in TBLASTN searches against assembled genome sequence scaffolds of pear genome using the same parameters. All BLAST hits together with 3,000 to 6,000 bp of flanking sequence were manually annotated to produce complete CDSs using the program of FGENESH on Softberry website (http://linux1.softberry.com/berry.phtml).

Pfam analysis was then used to verify the presence of NB-ARC domain and LRR motif in all candidates identified. The SMART protein motif analysis (http://smart.embl-heidelberg.de/) was performed to improve the accuracy and integrity of LRR identification. Subsequently, the NBS-LRR genes were determined by using the combination results of Pfam and SMART analysis. Finally, all sequences were analysed to further verify the presence of TIR or CC domain using Pfam and COILS (http://embnet.vital-it.ch/software/COILS_form.html) databases, which were used to classify the NBS-LRR genes into two sub-groups: TIR-NBS-LRR genes which contain TIR domain and non-TIR-NBS-LRR genes which contain CC or other (X) domains.

All-versus-all BLASTN searches were performed with an E-value of 1.0 across the NBS-LRR CDSs in the five Rosaceae species. Genes were divided into gene families based on (1) the cutoff of coverage larger than 70% (aligned sequence lengths/gene lengths), and (2) the identity between sequences exceeding 70%.

### Sequence alignment and phylogenetic analysis

The amino acid sequences of all NB-ARC domains were aligned using the MUSCLE program with default options in MEGA v5.0 [35]. Subsequently, a Maximum Likelihood (ML) method was used to construct the phylogenetic tree with Jukes-Cantor model of nucleotide evolution and 1,000 bootstraps through FastTree v2 [70]. For RPW8 domain-containing NBS-LRR genes, the alignments were obtained by the same method and used to construct the phylogenetic tree based on NJ method using pairwise deletion of gaps and kimura-2 model with 1,000 replicates in MEGA v5.0 [35].

### Estimation of nonsynonymous substitutions and synonymous substitutions

The nucleotide sequences of CDSs in each gene family were aligned based on the protein sequences by using Clustalw 2.0 [71]. The nonsynonymous substitutions (*Ka*) and synonymous substitutions (*Ks*) and nonsynonymous to synonymous substitution ratios (*Ka*/*Ks*) were estimated in each gene family according to the alignments in MEGA v5.0 [35].

### Test for selective pressures

The PAML4 [36] package was used to perform the site model and branch model test to detected selective pressures of NBS-LRR genes among five Rosaceae species. For the site model, one single *dN*/*dS* ratio (model = 0) and models M7 (beta) and M8 (beta-ω) (NS site = 7 8) were applied among all gene families containing three or more members. The LR test between model M7 and M8 was also performed by using critical value of chi-square 5.991 (*p* < 0.05, *df* = 2) and 9.210 (*p* < 0.01, *df* = 2). One single *dN*/*dS* ratio (model = 0) and models 0 (NS site = 0) were implemented using the codeml program for branch models.

## Additional files

Additional file 1: Table S1. Gene families of NBS-LRRs in the five Rosaceae fruit genomes. Additional file 2: Table S2. Nucleotide diversity of RPW8 domain-containing genes in each group. Additional file 3: Table S3.
*Ka*/*Ks* ratios of RPW8 domain-containing genes in each group. Additional file 4: Figure S1. Phylogenetic tree of NBS-LRR genes in strawberry genome. Fuchsia branches represent TNL genes and light blue branches represent non-TNL genes. Additional file 5: Figure S2. Phylogenetic tree of NBS-LRR genes in apple genome. Fuchsia branches represent TNL genes and light blue branches represent non-TNL genes. Additional file 6: Figure S3. Phylogenetic tree of NBS-LRR genes in pear genome. Fuchsia branches represent TNL genes and light blue branches represent non-TNL genes. Additional file 7: Figure S4. Phylogenetic tree of NBS-LRR genes in peach genome. Fuchsia branches represent TNL genes and light blue branches represent non-TNL genes. Additional file 8: Figure S5. Phylogenetic tree of NBS-LRR genes in mei genome. Fuchsia branches represent TNL genes and light blue branches represent non-TNL genes.

## Abbreviations

NBS-LRR: Nucleotide binding sites-leucine-rich repeats
*R* gene: Resistance gene
CDS: Nucleotide coding sequences
