# Supplementary figures and images for: Species-specific duplications driving the recent expansion of NBS-LRR genes in five Rosaceae species
Source: BMC Genomics. 2015 Feb 14;16(1):77. doi: 10.1186/s12864-015-1291-0 (PMC4336698; doi:10.1186/s12864-015-1291-0)

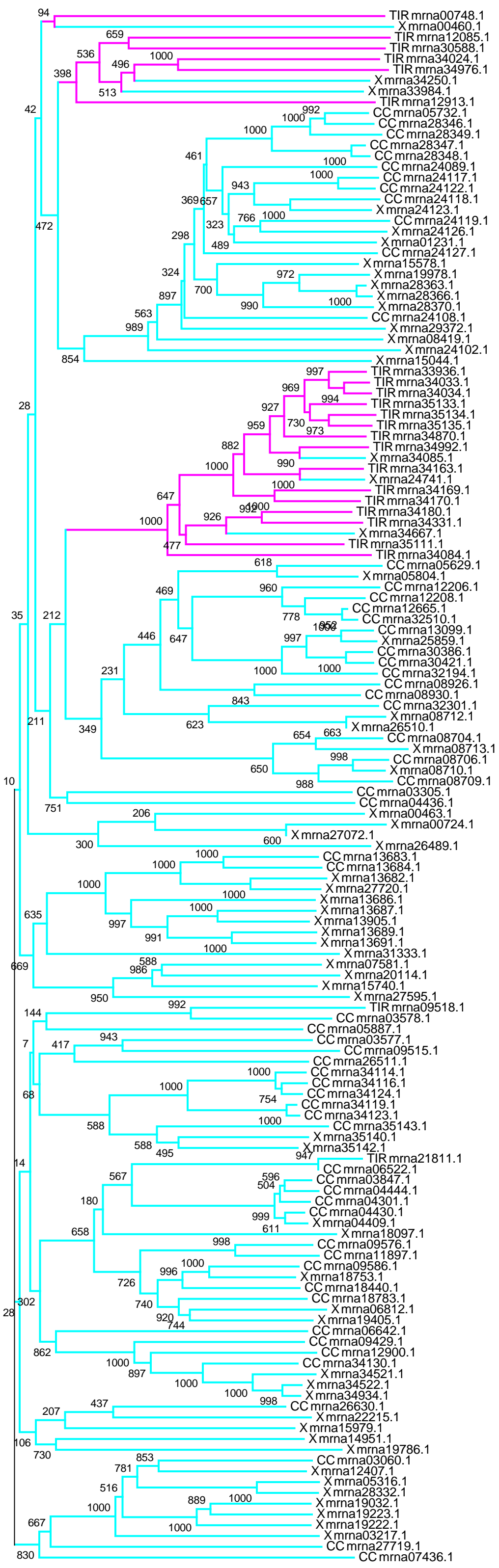

0.05

Supplement: Additional file 4: Figure S1. — Phylogenetic tree of NBS-LRR genes in strawberry genome. Fuchsia branches represent TNL genes and light blue branches represent non-TNL genes. [file 12864_2015_1291_MOESM4_ESM.pdf]

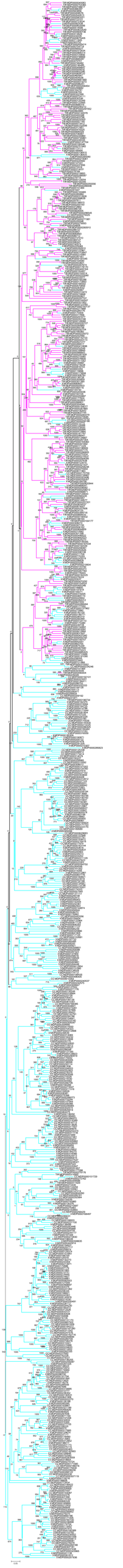

Supplement: Additional file 5: Figure S2. — Phylogenetic tree of NBS-LRR genes in apple genome. Fuchsia branches represent TNL genes and light blue branches represent non-TNL genes. [file 12864_2015_1291_MOESM5_ESM.pdf]

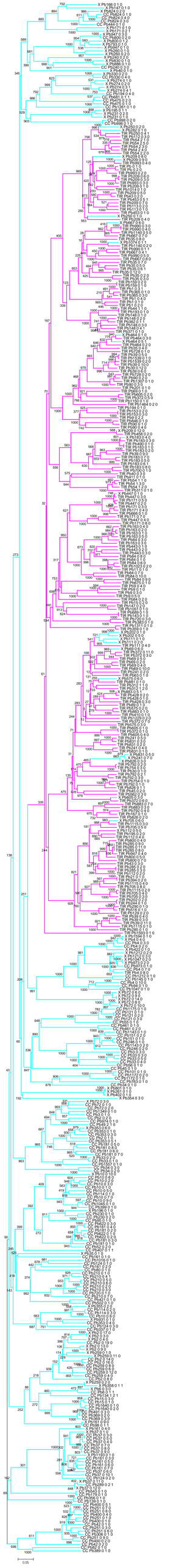

Supplement: Additional file 6: Figure S3. — Phylogenetic tree of NBS-LRR genes in pear genome. Fuchsia branches represent TNL genes and light blue branches represent non-TNL genes. [file 12864_2015_1291_MOESM6_ESM.pdf]

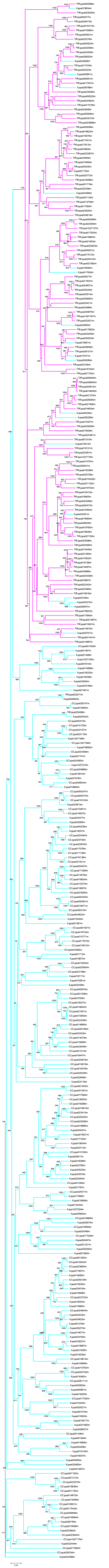

Supplement: Additional file 7: Figure S4. — Phylogenetic tree of NBS-LRR genes in peach genome. Fuchsia branches represent TNL genes and light blue branches represent non-TNL genes. [file 12864_2015_1291_MOESM7_ESM.pdf]
